# Stepwise Development of a Simulation-Based Musculoskeletal Ultrasound Curriculum for Physical Medicine and Rehabilitation Residents

**DOI:** 10.1177/23821205251370796

**Published:** 2025-08-19

**Authors:** Sarahrose Jonik, Terry Brosche, Michele Favreau, Paul Haidet, Neyha G. Cherin

**Affiliations:** 1 Penn State Health, Department of Internal Medicine, Penn State College of Medicine, Hershey, PA, USA; 2 Penn State Hershey Clinical Simulation Center, Hershey, PA, USA; 3 University of Hawai'i, John A. Burns School of Medicine, Honolulu, HI USA; 4 Penn State Health, Department of Medicine Division of General Internal Medicine, Penn State College of Medicine, Hershey, PA, USA; 5 Penn State Health, Department of Physical Medicine and Rehabilitation, Penn State College of Medicine, Hershey, PA, USA

**Keywords:** MSKUS, ultrasound, curriculum development, PM&R resident education, quality improvement, simulation-based medical education

## Abstract

**Objectives:**

Musculoskeletal ultrasound (MSKUS) is a required part of Physical Medicine and Rehabilitation (PM&R) residency training, yet there remains a lack of standardized educational framework guiding its implementation across residency programs. While structured MSKUS curricula exist, variability in institutional resources limits generalizability. Moreover, there has been comparatively less focus on the advantages of incorporating longitudinal, simulation-based learning, which has been proven to enhance skill acquisition, clinical confidence, and patient safety. This study aims to address gaps in MSKUS curriculum by presenting the development and evaluation of a longitudinal, simulation-based MSKUS curriculum tailored to an academic PM&R residency program.

**Methods:**

A longitudinal, simulation-based MSKUS curriculum was developed and implemented for PM&R residents at an academic institution from 2021 to 2025. The curriculum underwent iterative refinement each year in response to resident feedback, institutional needs, and evolving best practices. Core components included simulation-based scanning, near-peer instruction, standardized patients (SPs), and structured assessments aligned with progressive learning milestones. The curriculum designed was informed by Kern's six-step model and guided by Miller's Pyramid to support skill development from knowledge acquisition to clinical application.

**Results:**

Feedback from curriculum implementation demonstrated increased clinical confidence and competence in MSKUS skill. Residents highlighted the value of active learning, clinical relevance, and peer collaboration. Iterative refinements addressed logistical barriers and aligned content with learner progression across PGY levels.

**Conclusion:**

This simulation-based MSKUS curriculum provides an adaptable framework for PM&R residency programs. By emphasizing hands-on practice with SPs, team-based learning, and iterative feedback, it fosters skill development, mentorship, and sustainable ultrasound education applicable across diverse training environments.

## Introduction

Musculoskeletal ultrasound (MSKUS) is an essential diagnostic and therapeutic tool used daily by a variety of specialties. Physiatrists have capitalized on the importance of MSKUS and practice this safe and economical approach to address patient's functional impairments.^1^ In recognition of its growing role, the American Board of Physical Medicine and Rehabilitation (ABPM&R) updated residency graduation guidelines in 2015 to include exposure to 10-15 MSKUS procedures by graduation.^2,3^ Despite MSKUS being a core educational objective in PM&R training, the specialty has yet to identify a standardized MSKUS educational framework or set of core competencies. This stands in contrast to other specialties, like Emergency Medicine and Sports Medicine, which have implemented structured MSKUS curricula.^4,5^ In the absence of such guidance, PM&R programs are left to independently interpret how to deliver MSKUS education to their learners. Some programs emphasize image acquisition or interpretation, while others focus on clinical application. There is also a vast difference between the didactic approach to teaching; passive lecture versus hands-on education. These inconsistencies, along with the rapid evolution of ultrasound technology and its clinical integration, contribute to significant gaps in PM&R MSKUS education nationally.^6^ While PM&R programs in more densely populated locations have been more likely to create formal curricula,^7-9^ these models may be less readily transferable to rural or resource-limited settings, where differences in faculty availability, equipment access, and institutional resources present unique challenges.

There are documented barriers to implementing formalized MSKUS curricula in PM&R programs.^10^ A 2010 survey identified insufficient MSKUS training as a primary reason attending physicians did not incorporate MSKUS into their clinical practice.^10^ In a subsequent survey of PM&R program directors, only 44% reported having a formalized curriculum in their programs.^11^ A 2015 follow-up survey emphasized the importance of MSKUS training, identifying it as an integral component of residency education.^6^ That same year, the ABPMR revised residency guidelines to align with this national perspective, mandating exposure to 10-15 MSKUS procedures prior to graduation.^2,3^ Despite the growing recognition and importance of MSKUS, no accreditation guidelines were formalized to assist residency programs with implementing this new requirement. A 2019 survey further explored the challenges that persisted in providing MSKUS “exposure” to their PM&R trainees,^12^ with programs noting three significant barriers: a lack of standardized guidance on curriculum content, limited knowledge and experience among attendings, and inadequate equipment availability.^12^

As of 2025, 115 accredited PM&R residency programs exist nationwide,^13,14^ and enthusiasm for MSKUS is at an all-time high. Programs with MSKUS curricula have noted enhanced learning and anatomical palpatory accuracy.^15-20^ Beyond educational outcomes, the presence of a formal MSKUS curriculum has broader implications. For example, programs with formal MSKUS curriculum were found to achieve higher resident satisfaction scores.^7,8^ Additionally, a 2020 survey of medical students applying to PM&R found that applicants ranked programs with formal MSKUS curricula higher on their match lists.^21^

Recognizing the evolving needs of their learners, PM&R programs are restructuring MSKUS education to better prepare future clinicians for the demands of the field. Unfortunately, significant obstacles remain. This very question was explored at Penn State Health Milton S. Hershey Medical Center PM&R (PSHR) program. At its start in 2015, the PM&R residency program lacked a formalized MSKUS curriculum. In 2019, the author was tasked with creating and implementing a standardized MSKUS curriculum at PSHR. The curriculum, originally planned to begin in 2020, faced numerous challenges. As a small rural program, limited MSKUS faculty availability, time constraints, and insufficient department funding and resources posed substantial barriers. These issues were further exacerbated by the COVID-19 pandemic, which placed additional strain on the institutional capacity. Despite these barriers, the curriculum planning continued and was formally launched in 2021.

This article seeks to address current gaps in MSKUS curriculum by presenting the development and implementation of a structured, simulation-based, longitudinal MSKUS curriculum tailored to the needs of an academic PM&R residency program. By describing the rationale, structure, and outcomes of this curriculum, we aim to provide a reproducible educational model, guided in Kern's six-step framework, that equips learners with the skills needed to meet the evolving demands of modern Physiatric practice.

## Methods

The MSKUS curriculum was initiated in 2019, with its first implementation in the fall of 2021. Since its inception, the curriculum has completed four iterative cycles, evolving in response to learner feedback, program needs, and broader healthcare challenges, including the COVID-19 pandemic and ongoing national healthcare policy modifications. Kern's six-step approach to curriculum development served as a systematic framework, guiding the design, implementation, and continuous refinement of the curriculum.^22-24^ Each academic year, the six steps—1) problem identification and general needs assessment, 2) targeted needs assessment, 3) goals and objectives, 4) educational strategies, 5) implementation, and 6) evaluation and assessment—were revisited to ensure curriculum relevance, effectiveness, and feasibility. This educational quality improvement initiative conforms to the SQUIRE 2.0 guidelines^25^ [Supplemental File 1].

### Demographic

This curriculum serves PGY-2 (n = 4), PGY-3 (n = 4), and PGY-4 (n = 4) PM&R residents and medical students (MS1-MS4; n varies) at Hershey Medical Center, a tertiary academic medical center in Central Pennsylvania. One faculty member oversaw the teaching, assessment, and production of the curriculum. Given learners’ varied training and MSKUS exposure levels, the course emphasized adaptable, learner-centered goals. It's primary goal is to equip trainees with strong foundations in anatomy, physical examination, and MSKUS skills for clinical application.

#### Year 0 (2019-2021): Curriculum Design and Development

In 2019, the author and program leadership conducted a general needs assessment. Given that the residency program was established in 2015, anecdotal and qualitative feedback from 2017 to 2018 was utilized. A more targeted follow-up included informal surveys of residents, alumni, and MSKUS-trained faculty. Alumni reported seeking external continuing medical education courses to build their MSKUS skillset, while current residents expressed a desire for a structured, in-house training. Faculty agreed, identifying a clear educational gap and significant opportunity for growth.

Learning environment challenges were multifaceted. The program had limited MSKUS-trained faculty, who were also balancing full-time clinical responsibilities. Financial constraints and reliance on a single ultrasound machine further restricted training opportunities. Additionally, the program's rural setting limited access to more diverse patient populations and reduced opportunities for interdepartmental collaboration.

The department noted a clear disparity between the program's current MSKUS training approach and ABPM&R national standards and curricula implemented in larger PM&R programs.^5,7,21^ Faculty frequently employed the “see one, do one, teach one” approach with residents; however, the limited exposure to the musculoskeletal patient population at the time hindered residents from completing this three-tiered process and fully developing their ultrasound skills.

Between 2015 and 2019, interdepartmental efforts were trialed, including short-term electives; however, these were largely ineffective due to their unstructured nature, minimal hands-on experience, and competing demands from other residency programs at the academic institution. In response, the program encouraged self-guided practice, but learner feedback revealed this approach to be largely ineffective, noting time constraints, restricted machine access, and lack of faculty oversight. The COVID-19 pandemic further compounded these issues by limiting clinic access and procedural exposure for trainees.

### Curricular Framework and Evaluation Models

To support curriculum evolution, emphasis was placed on developing robust assessment tools. The evaluation phase incorporates feedback strategies to measure curricular effectiveness and monitor individual learner progress, consistent with Kern's six-step approach. This process evaluates not only knowledge and skill acquisition, but also the practical application of these competencies and their impact on clinical care.

#### Year 1 (2021-2022): Curriculum Implementation

Launched in 2021, the curriculum incorporated ongoing COVID-19 safety measures, including limiting participation to residents and hosting monthly sessions at the rehabilitation hospital to ensure a controlled and consistent learning environment. Each session focused on a particular joint, referenced in Table 1.

**Table 1.** MSKUS Curriculum Session Topics for Years 1-4.

Monthly	Session topic (Years 1-3)	Session topic (Year 4)
Session 1	Ultrasound Basics	Ultrasound Basics
Session 2	Wrist and Hand	Wrist and Hand
Session 3	Shoulder	Shoulder
Session 4	Elbow	Elbow
Session 5	Review Upper Body + Injection practice via phantom molds	Review Upper Body + Injection practice via molds
Session 6	Ankle	Ankle
Session 7	Hip	Hip
Session 8	Knee	Knee
Session 9	Lower Body Review + Injection practice via phantom molds	Lower Body Review + Injection practice via molds
Session 10	Curriculum Review	Objective Structured Assessment of Ultrasound Skills (OSAUS)

MSKUS, musculoskeletal ultrasound.

### Learning Objectives

Recognizing the foundational role of anatomy and physical exam skills in PM&R, and that learner confidence improves when anatomy is taught alongside ultrasound,^26,27^ the curriculum initially prioritized anatomical proficiency. A key objective was to strengthen learners’ anatomical acumen, which have evolved to reflect the program's adaptable and iterative structure.

The learning objectives for each session included the following:

Based on the selected joint:

1. Demonstrate the appropriate physical examination test relevant to the anatomy.
2. Articulate the indications and appropriate MSKUS technique.
3. Assess for normal and abnormal sonographic appearances of tissue, nerve, muscle, tendon, bone, and vasculature, using European Society of Musculoskeletal Radiology (ESSR) guidelines.
4. Employ near-peer teaching to create a collaborative environment where constructive feedback enhances understanding of MSKUS techniques and their applications.

### Year 1 Structure

To emphasize anatomical review and hands-on learning, learners were divided into four mixed-level groups (PGY2–PGY4), rotating through four stations with integrating the educational strategy of near-peer teaching. To support active learning without overwhelming residents, optional pre-session readings are provided. These resources encourage engagement by allowing learners to explore content in advance and lead discussions.

1. Activity 1: Joint specific hands-on MSKUS training, supervised by PM&R faculty.
2. Activity 2: Learner led anatomy-based clinical case discussions, fostering near-peer teaching and diagnostic reasoning.
3. Activity 3: Anatomy PhD student facilitated targeted anatomical review, reinforcing clinical correlations.
4. Activity 4: Musculoskeletal radiologist-led multimodal imaging interpretation instruction (MRI, radiographs, and ultrasound).

Evidence-based educational strategies shaped the course timeline to optimize learning and retention. Research supports spaced, repetitive hands-on practice to enhance knowledge acquisition, critical thinking skills, and long-term retention.^28-30^ Active, hands-on learning enhances the development of critical thinking skills necessary for real-world practice.^31^ Unlike traditional didactics, our 12-month curriculum includes monthly 3-h sessions, allowing residents to complete it three times during residency, reinforcing skills through repetition and progression.

To offset limited faculty availability, this curriculum adopted near-peer teaching, a proven efficacious educational strategy that fosters collaboration, self-regulation, and confidence among learners.^32-34^ This approach allows residents to take on increasing responsibility as they progress through the program, transitioning from learners to educators with increasing confidence and competence. By engaging in teaching and mentoring roles, residents enhance their critical thinking, clinical knowledge, and leadership skills.

#### Year 2 (2022-2023): Iterative Refinement and Expansion

At the start of Year 2, limited instructor availability and the loss of anatomy and radiology support prompted a strategic collaboration with the Penn State Health Simulation Center (SIM). This partnership became instrumental in sustaining and expanding the curriculum by offering critical resources, including access to five US machines of various models, simulation tools, five standardized patients (SPs) with dedicated examination rooms, and facilities for case-based learning (CBL). Additionally, the SIM Center's audio-visual system enabled real-time observation and feedback, enhancing teaching effectiveness. The inclusion of SPs added realism, providing residents with constructive feedback on patient positioning, communication, and professionalism in a safe, supportive environment.

### Modified Year 2 Structure

Anatomy-based discussions evolved to CBL exploring anatomy, diagnostic principles, and ultrasound techniques tailored to the joint of focus for the session. Learners, including both PGY-2 to PGY-4 residents and any medical student learners who were rotating with the department, analyzed clinical case vignettes, proposed diagnoses, and identified relevant anatomy and physical exam techniques. These sessions emphasized collaborative reasoning and knowledge synthesis, which was supported by board-relevant instruction delivered by the faculty facilitator, ensuring that learning objectives align.

Following CBL, residents were divided into four mini groups consisting of at least three learners, one PGY-2, one PGY-3, and one PGY4 (+/– rotating medical students). Each group practiced an assigned ultrasound skill with a SP and US machine, then taught the larger group, promoting peer teaching, active learning, and leadership. The faculty facilitator oversaw the teaching of the four groups to ensure accuracy, provide feedback, and reinforce alignment with ESSR guidelines.

#### Year 3 (2023-2024): Simulation Best Practices

Year 3 marked a pivotal phase with a focus on simulation best practices. The Simulation Center's educator attended sessions to ensure the application of evidence-based simulation practices, including structured prebriefs to establish psychological safety, guided debriefs to foster reflective learning, and optimized use of SPs and simulation technology.

Simulation provides a safe, controlled environment for learners to practice complex problem-solving, critical thinking, and technical skills without patient risk.^35^ This approach has been shown to enhance learner preparedness for clinical practice, fostering reflection, independence. and confidence, while empowering residents to manage patient interactions in real-time across a variety of settings.^36-38^ In essence, the simulation environment actively integrates theoretical learning with hands-on experience and structured reflection, making it a powerful tool for developing critical thinking and clinical judgment.

The course director and simulation educator co-managed sessions, revised objectives, updated materials, led debriefs, and gathered performance data to guide ongoing curriculum improvement, in alignment with Kern's learner-centered model.

### Refined Learning Objectives

Updated learning objectives include the following:

For a specific joint:

1. Articulate the indications and appropriate MSKUS technique.
2. Compare and contrast normal and abnormal sonographic appearances of tissue, nerve, muscle, tendon, bone, and vasculature on a SP, using ESSR guidelines.
3. Critique and provide constructive feedback on integrating MSKUS on a SP.
4. Employ near-peer teaching and constructive feedback to enhance understanding of MSKUS techniques and their applications.
5. Reflect on session learning through a structured debrief.

### Modified Year 3 Structure

Session timelines adjustments are reflected in Table 2.

**Table 2.** Year 3 Timeline and Tasks for Each Session.

	Tasks per session
8:00 −8:05 AM	Prebrief: Prepares the learners and helps to create a psychologically safe environment.
8:05-8:20 AM	Task 1a: 15 question board-style quiz completed independently.
8:20-9:00 AM	Task 1b: Review quiz in groups utilizing near-peer teaching.
9:10-10:15 AM	Task 2a: Simulation-based MSKUS practice with SPs.
10:15-10:45 AM	Task 2b: Mini groups teaching large group specific MSKUS skills.
10:45-11:00 AM	Debrief: Gather, Analyze, Summarize (GAS) Structured Debriefing Technique.

MSKUS, musculoskeletal ultrasound; SP, standardized patient.

### Pre-briefing

Pre-briefing was standardized to prepare learners for the simulation environment and establish psychological safety, a foundational principle in simulation education.^39-41^ Sessions began by reviewing objectives, addressing behavioral expectations, introducing the environment, and logistical details. Facilitators emphasize the “basic assumption” that all learners are well-intentioned and striving to do their best. The prebrief also provides additional safety measures including confidentiality agreements and the use of a designated safety word for participants, fostering a supportive and inclusive learning environment.^39^

### Case-based learning to Quiz-Based Peer Review

In response to Year 2 feedback, CBL format was restructured into a joint specific board-style quiz. Learners complete the quiz independently, then collaboratively review answers in small groups, discussing clinical rationale for both correct and incorrect answers. This approach reinforced key anatomical and clinical concepts while promoting peer-to-peer teaching and critical thinking.

### Debriefing

Each session concluded with a Gather, Analyze, Summarize (GAS) structured debriefing technique.^42^ Debriefing is a cornerstone of the simulation curriculum, guiding learners through metacognitive reflection on their technical performance, adherence to ESSR scanning protocols, communication with SPs, and overall bedside manner.^42^ This process allows learners to identify strengths, weaknesses, and strategies for applying their learning in future clinical encounters, which is essential for clinical thinking.^43^ Faculty serve as content experts during debriefs, offering feedback and bridging simulation experiences with real-world applications.

### Year 4 (2024-2025): Advancing Evaluation and Curriculum Feedback

The curriculum at present integrates a variety of evidence-based strategies:

1. Pre-briefing
2. CBL and board-style quizzes
3. Near-peer teaching
4. Simulation-based learning
5. Structured debriefing
6. Summative Observed Structured Assessment of Ultrasound Skills (OSAUS)

By combining these strategies, the curriculum offers an immersive, learner-centered experience that emphasizes reflection, collaboration, and mastery of MSKUS.

### Modified Year 4 Structure

#### Pre-Curriculum and Post-Curriculum Assessments

At the start of Year 4, learners were tasked with completing a 20-question formative, pre-curriculum assessment designed to evaluate residents’ baseline ultrasound knowledge and technical skills. This assessment provided objective insight into both individual and cohort-specific learning needs, allowing tailored instruction throughout the program. Upon completing the curriculum in 2025, residents will complete a corresponding 20-question formative, post-curriculum assessment to evaluate residents’ knowledge acquisition and practical skill development. As this component is newly integrated into the 2024-2025 curriculum, the first round of data analysis will be conducted at the year's end.

#### Peer Leadership

Given the transformative impact of simulation-based prebriefs and debriefs, senior residents began leading these sessions. This reinforced near-peer teaching, promoted leadership, and fostered community and continuity in educational practices.

#### OSAUS

The 2024-2025 curriculum introduced an annual OSAUS using SPs to assess ultrasound proficiency, including image acquisition, clinical integration, and communication. This validated assessment framework design evaluates ultrasound proficiency across multiple domains and has been recognized as an effective tool for assessing progress in ultrasound education within medical training programs.^44,45^

Residents will complete one of three standardized, real-world cases aligned with session topics to evaluate MSKUS skills, professionalism, and clinical reasoning. These cases are designed to reflect real-world scenarios and assess the application of foundational skills in MSKUS as well as professionalism, communication, and clinical reasoning. This assessment will allow for appraisal of residents’ growth and competence in ultrasound skills from PGY2 to PGY-4. Residents will obtain written and oral feedback from observing faculty and SPs. This will occur annually at the culmination of the 12-month program, allowing residents to track their progress over time and identify specific areas for continued development.

#### Year 5 (2025-2026): Future Direction

Planning for Year 5 is already in progress. In response to the evolving landscape of healthcare and advances in extended reality, the curriculum seeks to integrate cadaver-based virtual reality (VR) models to deepen anatomical understanding. Additionally, in collaboration with the SIM Center Design Team and the Harrell Library staff, we have developed 3D life-like joint molds to support hands-on practice of ultrasound-guided interventions. We will have learners perform injections of various joints using VR with the 3D models. Of note, given ongoing national health policy changes, we actively monitor and adjust our curriculum accordingly. These innovations aim to further enrich the educational experience and expand the scope of the longitudinal MSKUS curriculum, ensuring its continued evolution to meet the needs of modern PM&R practice.

## Results

A total of four annual cycles (2021-2025) of the MSKUS curriculum were implemented and iteratively refined. Table 3 summarizes key changes by year, reflecting adaptations in response to learner feedback, resource availability, and simulation best practices.

**Table 3.** Evolution of MSKUS Curriculum Based on Learner Feedback.

Academic year	Evolution of MSKUS curriculum
Year 0 (2019-2021)	The author initiated an examination of MSKUS opportunities within the health system and began developing a dynamic, adaptable curriculum in response.
Year 1 (2021-2022)	**Total of 10 sessions** o **Activity 1:** Review of 2D and 3D anatomy led by Anatomy PhD students. o **Activity 2:** Review of musculoskeletal clinical cases led by senior residents. o **Activity 3:** Review of MSKUS with European Society of Musculoskeletal Radiology (ESSR) diagnostic guidelines and protocols led by course director. o **Activity 4:** Review of advanced imaging led by musculoskeletal radiologist. **Barriers:** o COVID-19 safety measures o Location limited to rehabilitation hospital o 1 shared ultrasound machine split between clinic patients and resident didactics o 1 PM&R faculty available
Year 2 (2022-2023)	**Total: 10 sessions** o Location available at Penn State Hershey simulation center (6 patient rooms). o 6 freely available ultrasound machines o 6 standardized patients. **Barriers:** o No Anatomy PhD students available o 1 PM&R faculty available
Year 3 (2023-2024)	**Total: 10 sessions** o Integrated Simulation Nurse Educator to aide with pre-briefing and debriefing standards to enhance experiential learning. o Incorporated the near-peer teaching method, with seniors acting as faculty o Integrated nerve and muscle site review commonly used in clinical practice. **Barriers:** o No Anatomy PhD students available o 1 PM&R faculty available
Year 4 (2024-2025)	**Total: 10 sessions** o Incorporation of OSAUS at the culmination of each year. o Inclusion of board-style relevant questions and problem-based learning cases to each session. o Strengthened near-peer teaching with PGY-4's mentoring junior residents.
Year 5 (2025-2026)	New health policy considerations Integrated artificial intelligence and virtual reality into the curriculum to enhance experiential learning and clinical skill development.

MSKUS, musculoskeletal ultrasound; PM&R, Physical Medicine and Rehabilitation.

### Kirpatrick Model and Miller's Pyramid

**Figure 1.**
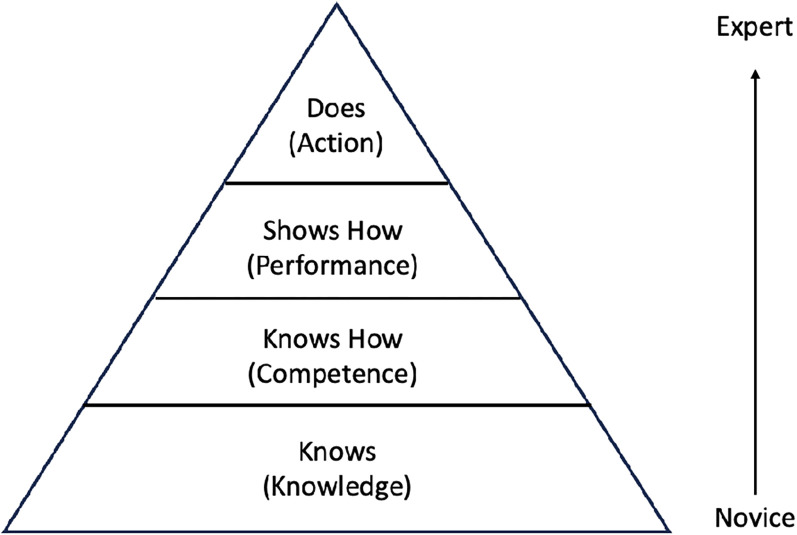
Representation of George Miller's Pyramid of Clinical Competence. Adapted from George Miller's Framework.

To guide our assessment, we employed Miller's Pyramid of Clinical Competence and the Kirkpatrick Model of Training Evaluation, ensuring a comprehensive analysis of learner outcomes and curriculum impact.^46^ Miller's Pyramid is a framework used to assess residents’ clinical competence at four levels: knows, knows how, shows how, and does (Figure 1). Each level represents a higher level of competence and skill development, with most assessment occurring in the intermediate levels (knows how and shows how*)* as residents apply MSKUS knowledge and skills through peer teaching, hands-on practice, and structured assessments. This repeated exposure fosters skill reinforcement and progression as residents advance from PGY-2 to PGY-4.^47^

**Figure 2.**
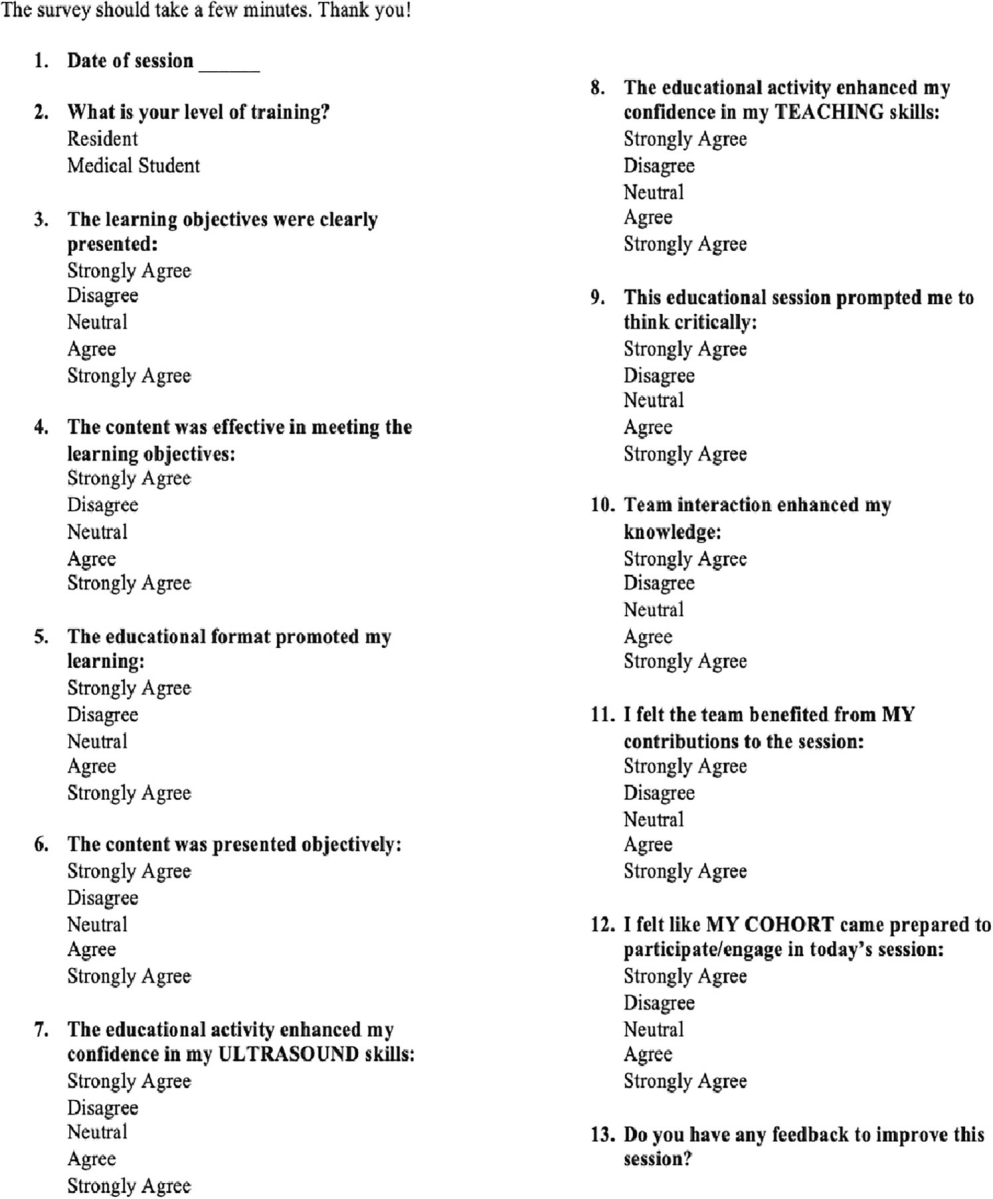
Session Feedback Survey Utilizing Both Likert-Scale Items and Open-Ended Questioning.

The Kirkpatrick Model evaluates training across four levels: reaction, learning, behavior, and results.^48^ At level 1 (reaction), resident's perceptions are gathered through post-session anonymous surveys (Figure 2). The survey collects participant feedback on session objectives, content delivery, teaching confidence, critical thinking, team collaboration, and engagement. These surveys highlight strengths, guide improvements, and assess unique curriculum features such as peer teaching, anatomy-MSKUS integration, real-time feedback, and the benefits of annual repetition for clinical application.

At level 2 (learning), the pre- and post-curriculum assessments evaluate residents’ knowledge acquisition, skill development, and changes in confidence, providing measurable evidence of their progress.

Learner feedback collection evolved over the years. In the first year, feedback was collected through open-ended, subjective feedback cards that were completed at the end of each session. Resident feedback in the second and third year was collected via a more comprehensive paper-based survey distributed to learners at the end of each session, which included both subjective, open-ended questions and objective Likert-scale items. Due to the format and timing of administering the survey, there was a noticeable decrease in open-ended responses compared to Year 1. A key curricular modification based on prior feedback was the introduction of CBL and the integration of the simulation best practices and SPs. Feedback for the current year was collected via a QR code-linked survey administered at the conclusion of each session. The survey included mandatory Likert-scale questions and open-ended responses.

### Qualitative Review

Learner feedback was collected annually over the 4-year implementation of the curriculum using a combination of open-ended responses and Likert-scale surveys. Across all years, participants included all residents in the training program (n = 12 with 4 residents per PGY cohort) as well as a total of 19 medical students, with student participation varying annually based on rotation schedules. A cumulative total of 226 open-ended comments were obtained, in addition to responses to the structured Likert-Scale questions. In year 1, feedback was optional and submitted solely by residents. In year 2, feedback collection was standardized and required at the conclusion of each session, yielding the highest response volume. In years 3 and 4, participation reverted to an optional format. Due to variability in feedback collection methods and response rates across years, a qualitative synthesis of repetitive learner comments is presented below.

Multiple themes emerged based on learner feedback, summarized in Table 4.

**Table 4.** Summary of Feedback from Years 1-4 with Associated Themes.

	Theme	Year 1 (2021-2022)	Year 2 (2022-2023)	Year 3 (2023-2024)	Year 4 (2024-2025)
1	Hands-on, active, and peer-based learning	Valued hands-on US challenges, peer scanning, and group interaction.	Described sessions as educational and practical; valued interactive learning.	Strongly endorsed peer-led teaching, teach-back format, and collaboration.	Expressed increased confidence in teaching skills; valued team interaction.
2	Structured guidance and technical skill development	Requests for improved scanning technique and machine use guidance by leading faculty.	Desire for clearer roles and expectations during SP encounters.	Requests for attending-led instruction before small group practice.	Structured feedback through GAS debriefs helped build US skills.
3	Clinical relevance and curriculum structure	Appreciated board-style questions and clinical correlation.	Appreciated high-yield content; sessions described as useful for clinical practice.	SIM and board-based scenarios viewed as clinically impactful and relevant.	Improved confidence in critical thinking and clinical application over time.

SP, standardized patient; GAS, Gather, Analyze, Summarize.

### Theme 1: Value of Hands-on, Active, and Peer-Based Learning

Across all years, residents emphasized the importance of interactive, hands-on learning and peer-led instruction. They appreciated ultrasound challenges, teach-back formats, and near-peer teaching as strategies that deepened understanding and engagement.

- Year 1: Requests for more hands-on time and interactive sessions; peer scanning and real-time case discussion were valued. For example: “Increase frequency if possible of US education.”
- Year 2: Sessions were consistently described as “educational” and “practical.”
- Year 3: Strong praise for peer-led teaching, group learning, and simulation-based practice. For example: “Excellent content. Really like the teaching rounds with each group.”
- Year 4: Learners noted growing confidence in teaching skills and highlighted the value of team-based knowledge development.

### Theme 2: Desire for More Structured Guidance and Technical Skill Development

Residents repeatedly requested more structured ultrasound instruction, including machine handling, probe positioning, and expert modeling of scanning techniques.

- Year 1: Emphasis on deepening technical US skills and recognizing anatomy with requests for more guidance on machine use. For example: “More practice with physical exam, special tests, and clinical correlates.” And “Would like to use more time to scan or learn technical aspects of machine.”
- Year 2: Need for clearer expectations during SP encounters and structured session flow. For example: “It would be helpful to clarify with the SPs beforehand the extent to which the exercise is a patient encounter versus and US practice session.” And “Make clear guidelines/expectations to patients that this is an educational ultrasound session.”
- Year 3: Requests for more attending-led demonstrations before peer group practice. For example: “I want to continue to learn from MSK radiology.” And “Prefer direct attending teaching/guidance to show US first.”
- Year 4: Increased confidence in US skills reported, though early sessions still required structured setup (eg, through GAS debriefs). For example: “This US course is very educational and helpful as a resident interested in using US in practice.”

### Theme 3: Clinical Relevance and Curriculum Structure

Learners consistently valued the curriculum's clinical relevance, especially when tied to board-style questions, real-world scenarios, and opportunities for reflection through simulation and structured feedback.

- Year 1: Appreciation for board-style questions and clinical correlation; desire for increased frequency. For example: “Good integration of board questions.”
- Year 2: High-yield clinical content and usefulness for future practice noted. For example: “Enjoyed cases in the beginning with board-relevant information and questions.”
- Year 3: SIM, SPs, and case-based boards viewed as clinically impactful and engaging. For example: “Enjoyed cases in the beginning with board-relevant information and questions.”
- Year 4: Curriculum enhancements (eg, OSAUS, team-based debriefs) showed improved confidence in critical thinking and skill application. For example: “Safe learning environment.” And “Enjoyed reviewing topics as a group.”

**Figure 3.**
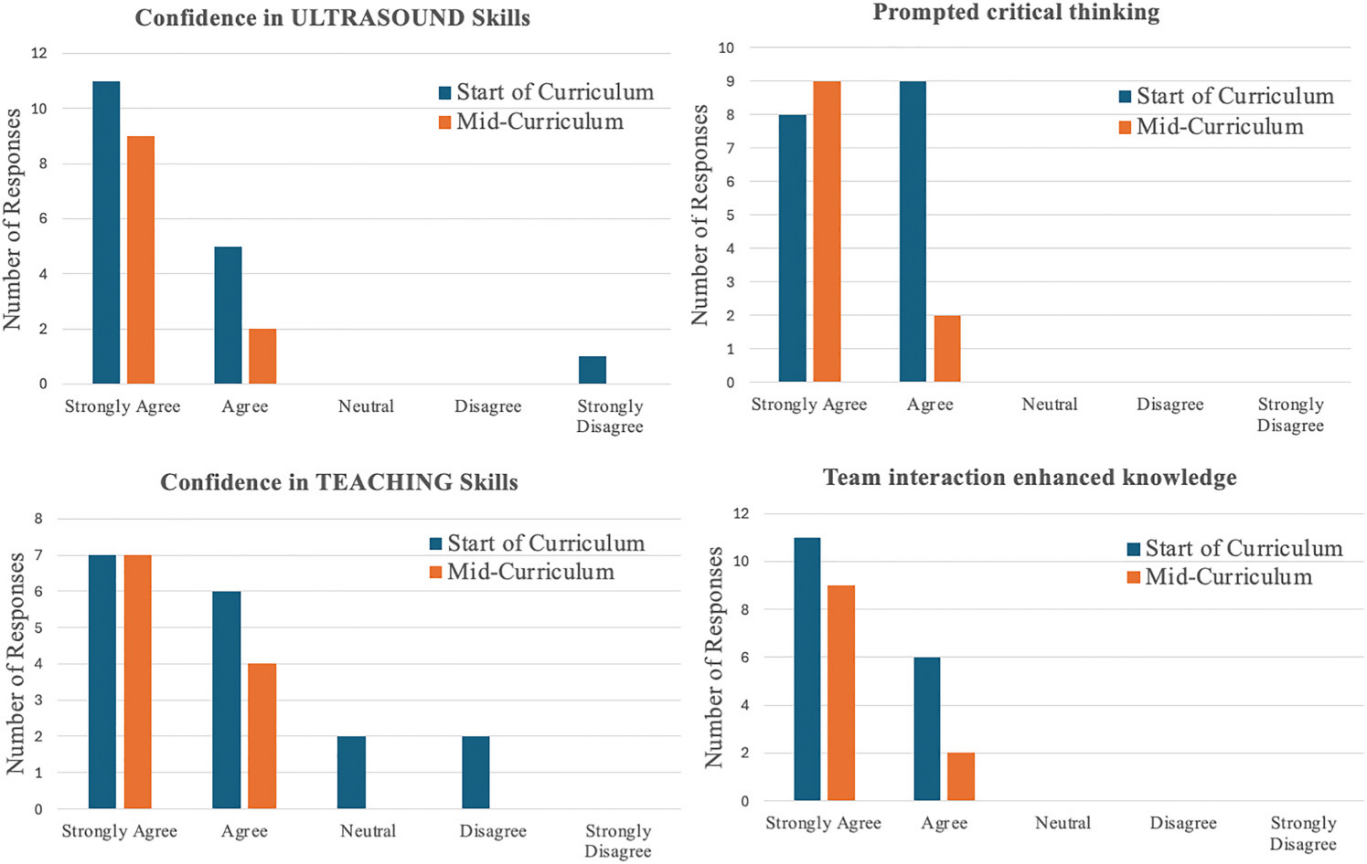
Comparison of Year 4 MSKUS Feedback: Start Versus Mid-Curriculum. Learner Responses Comparing Perceptions at the Start (n = 17y) and Midpoint (n = 11) of the Year 4 MSKUS Curriculum. MSKUS, Musculoskeletal Ultrasound.

Figure 3 demonstrates Year 4 feedback from learners (n = 17 for first session, n = 11 for mid-curriculum) to assess resident perception on: confidence in ultrasound skills, confidence in teaching skills, critical thinking development, and the value of team interaction on knowledge. Each used a 5-point Likert-scale. The data shows a positive trajectory in learner experience and perceived competence as the curriculum progresses. Confidence in both ultrasound and teaching skills improved, with learners also placing increased value on critical thinking and team collaboration.

Ultimately, while objective evidence of patient outcomes or long-term clinical impact is not yet available, anecdotal reports suggest that the curriculum contributes to residents’ readiness to use MSKUS effectively in their professional careers. This combination of qualitative and quantitative assessments ensures a comprehensive evaluation of the curriculum's success in achieving its goals.

## Discussion

This project aims to address gaps in MSKUS curriculum development for PM&R residency programs. The article illustrates how Kern's six-step framework was utilized to create and implement a hands-on, longitudinal, simulation-based PM&R MSKUS curriculum while highlighting the importance of maintaining a structured framework which allows curriculum evolution to meet the evolving demands of modern physiatric practice. Ultimately, the goal is to offer an adaptable and sustainable MSKUS educational model that responds to evolving learner needs, addresses resource variability, and supports high-quality, competency-based physiatric training. To the author's knowledge, this is the first study exploring the use of simulation and SPs in a longitudinal MSKUS training model specifically tailored to the needs of PM&R trainees.

### Strengths and Limitations

The curriculum demonstrates several key strengths. It leverages near-peer teaching, GAS debriefing techniques, and modular, flexible design to foster technical competency, reflective learning, and progressional growth in leadership and communication. Developed without dedicated funding, it utilizes existing institutional resources and aims to serve as an adaptable model adaptable to a wide variety of training environments.

The integration of near-peer teaching reinforces core concepts, promotes learner autonomy, and fosters the development of essential teaching skills. Residents received training in GAS debriefing techniques and were subsequently given opportunities to lead debriefing sessions, thereby enhancing reflective learning. In parallel with MSKUS skill development, the curriculum also cultivated leadership and teaching capabilities, key components of an academic training environment. Learners consistently highlighted the strong sense of community and psychologically safe learning environment fostered by these elements. Near-peer teaching facilitates collaboration, reduces reliance on faculty, and supports a sustainable educational pipeline, especially critical in resource-limited environments. At our institution, where PM&R faculty with ultrasound expertise are limited due to a neuro-heavy focus, this model has proven to be the most efficient use of faculty time when one person is tasked with leading curriculum design and implementation. While collaborative models with other medical specialties could also provide a platform for shared resources and training, competing department goals and educational objectives often can diminish these types of efforts. Interprofessional training, however, can be a bonus for all learners, as sharing different perspectives on patient treatment and care can potentially improve patient outcomes.

The curriculum's modular structure allows for flexibility. In response to learner and program feedback, objectives were modified: initially emphasizing anatomical skills to match cohort experience and progressively shifting toward more advanced learning goals. Educational strategies were deliberately adapted over time, including the introduction of CBL to promote active engagement, along with providing a virtual platform if necessary. Monthly sessions permit dynamic cohort sizes, and the structure easily accommodates varying group numbers. Its hands-on, in-person training enables residents to develop technical competency and spatial understanding through direct interaction with varying ultrasound equipment and SPs.

Aspects of this program can also be applied to under-resourced settings such as rural sites. For example, this model can create teaching efficiencies for a singular faculty member in a rural or other under-resourced setting by directly engaging resident trainees in the educational process through peer teaching, direct observation, and provision of meaningful feedback to trainees. Coupled with self-directed learning strategies, peer teaching can significantly reduce the educational burden for faculty and enhance the overall learning experience for all participants. With possible financial support from grants, the health system, and/or the university, SPs can be recruited and used within available clinical space to afford residents useful practice in communication and clinical decision-making skills, with even limited ultrasound technology. For example, organizations such as the Society for Simulation in Healthcare offer free resources to support simulation-based education in low-resource settings. Additionally, procuring institutional and/or grant support can continue to support innovative educational efforts that are adaptable, efficient, and ultimately sustainable across multiple educational environments.

Furthermore, as the health system financial and regulatory landscape continues to change and evolve, developing educational programs with efficiencies that can adapt to these changes is essential. The proposed MSKUS curriculum pairs self-directed, adult learning strategies with near-peer teaching sessions to create sustainable educational approaches to MSKUS training in the absence of teaching resources. Engaging community members as SPs and utilizing available clinical space to enable trainees to “practice” and hone their technical and communication skills is an effective use of limited resources to advance the trainees’ educational goals. These types of educational adaptations are not only valuable in terms of health system changes but can also be applied to other unforeseen resource restricted learning environments, such as the COVID 19 pandemic.

Despite its strengths, the curriculum has limitations. The most significant limitation is scalability, as delivering and maintaining a curriculum of this scope becomes increasingly difficult when led by a single faculty member without dedicated administrative support or protected time. Additionally, variability in faculty expertise can require interdepartmental collaboration, which can limit the generalizability of the curriculum. Its success also largely hinges on continuous real-time adaptation based on feedback, which is labor-intensive, and any delays can disrupt the schedule. Even though this model promotes the most effective use of faculty time and effort, it also requires constant overview and review of the curriculum to ensure the activities and stated learning objectives are consistently meeting the needs of the learners. This can require additional “cognitive” effort by the supervising faculty.

Currently, while the curriculum demonstrates strong learner engagement and reported improvements in knowledge and skills, it has not yet reached Kirkpatrick's Level 3 evaluation (behavioral change). Active efforts are underway to collect and analyze evaluation data that address this gap.

### Future Direction

Future steps for the MSKUS curriculum will focus on enhancing the curriculum's educational scope, evaluation metrics, and longitudinal outcomes. To further develop residents’ emotional intelligence skills, we will work to incorporate emotionally charged simulated cases, providing opportunities to practice empathy, self-awareness, and emotional regulation, which are key components of effective patient care. Additionally, OSAUS results will be systematically analyzed to provide objective data to track objective improvement in resident performance over the course of the curriculum. Pre- and post-test measures will be expanded to include larger sample sizes across multiple years, further validating the program's efficacy through objective data.

Faculty evaluations in clinical settings will be implemented to assess residents’ real-world skill application and evolution with clinic patients. Notably, as technology continues to advance, future studies could research the utility and efficacy of incorporating VR simulation to further enhance interactive learning, allowing residents to practice MSKUS techniques in an immersive, dynamic environment.

Long-term goals of this project include collecting self-reported feedback from recent graduates to evaluate their confidence and independence in MSKUS practice, assessing whether the curriculum adequately prepared them for independent patient care. Additionally, expanding the program to include additional residency cohorts will allow for more robust data analysis, highlighting trends and areas for refinement. These steps aim to further optimize the curriculum, ensuring it evolves to meet the needs of both learners and the dynamic demands of clinical practice.

## Conclusion

MSKUS is a critical component of PM&R residency education, yet the absence of standardized guidelines poses significant challenges for structured implementation. The PSHR program sought to address this gap by developing an adaptable curriculum tailored to the needs of an academic PM&R residency program. Its focus on active learning, fostering critical thinking, self-directed learning, and mentorship at all levels of training, creating a sustainable and collaborative educational environment. Using Kern's six-step approach and incorporating simulation-based learning, the curriculum emphasizes prebriefs, experiential learning through case-based discussions, hands-on ultrasound practice with SPs, near-peer teaching, and structured debriefs. Real-time modifications allow the curriculum to adapt to the evolving needs of learners, patients, and the healthcare system. This dynamic, learner-centered approach is designed to progressively build residents’ confidence and competence, ensuring they graduate with the skills required to independently and accurately perform MSKUS. Through continuous feedback and iterative refinement, this curriculum demonstrates a model for achieving optimal educational outcomes, preparing PM&R residents to confidently integrate MSKUS into their clinical practice and meet the evolving demands of the healthcare system. Rather than adhering to a one-size-fits-all model, we advocate for a dynamic, adaptable curriculum that evolves in response to environmental restrictions, learner feedback, cohort skill levels, and program goals. This learner-centered approach ensures relevance, engagement, sustainability, and maximal educational impact.

## Supplemental Material

sj-pdf-1-mde-10.1177_23821205251370796 - Supplemental material for Stepwise Development of a Simulation-Based Musculoskeletal Ultrasound Curriculum for Physical Medicine and Rehabilitation Residents Supplemental material, sj-pdf-1-mde-10.1177_23821205251370796 for Stepwise Development of a Simulation-Based Musculoskeletal Ultrasound Curriculum for Physical Medicine and Rehabilitation Residents by Sarahrose Jonik, Terry Brosche, Michele Favreau, Paul Haidet and Neyha G. Cherin in Journal of Medical Education and Curricular Development
